# Long-term decline in grassland productivity driven by increasing dryness

**DOI:** 10.1038/ncomms8148

**Published:** 2015-05-14

**Authors:** E. N. J. Brookshire, T. Weaver

**Affiliations:** 1 Department of Land Resources and Environmental Sciences, Bozeman, Montana 59715, USA; 2 Department of Ecology, Montana State University, Bozeman, Montana 59715, USA

## Abstract

Increasing aridity and drought severity forecast for many land areas could reduce the land carbon (C) sink. However, with limited long-term direct measures, it is difficult to distinguish direct drying effects from counter effects of CO_2_ enrichment and nitrogen (N) deposition. Here, we document a >50% decline in production of a native C_3_ grassland over four decades and assign the forcing and timing to increasing aridity and specifically to declining late-summer rainfall. Analysis of C and N stable isotopes in biomass suggests that enhanced water use efficiency via CO_2_ enrichment may have slightly ameliorated the productivity decline but that changes in N had no effects. Identical declines in a long-term snow-addition experiment definitively identified increasing late-summer dryness as the cause. Our results demonstrate lasting consequences of recent climate change on grassland production and underscore the importance of understanding past climate–ecosystem coupling to predicting future responses to changing climate.

## Abstract (web summary)

How primary production is influenced by climatic forcing has not been tested in most ecosystems. Here, the authors study a four-decade record of grassland production and find a sustained decline in above-ground net primary production attributable to increased aridity from declining late-summer rainfall.

## Graphical abstract

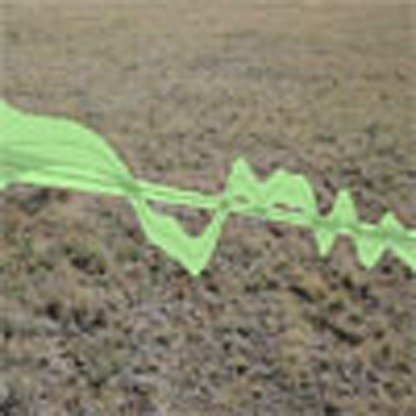

The extent to which past ecosystem responses to climate variation can predict those in the future depends on whether climate variation and ecosystem functions are deterministically coupled. Although climate variation clearly influences vegetation dynamics at local to global scales^1^,^2^,^3^,^4^, causal attribution to climate may be obscured by other forcing such as increased CO_2_ and N deposition. Increases in net primary production by land plants in response to rising CO_2_, for example, requires that the availability of water and other resources keep pace with increasing evaporative demand due to warming^5^,^6^. Recent global change experiments^5^,^6^,^7^,^8^,^9^ simulate future conditions via step changes in key drivers but do not simulate historical sensitivities to background-realized rates of variation. For example, although such experiments can simulate the effects of short-term departures from regional norms in precipitation (drought), it is far more difficult to simulate effects due to longer-term (decadal) trends in background aridity. Increases in drought severity and overall dryness are projected for many land areas globally^10^,^11^,^12^,^13^. How primary production is coupled to these different modes of climate variation over the last several decades of anthropogenic forcing remains empirically untested for most ecosystems.

We studied a four-decade (1969–2012) record^14^,^15^ of grassland production within the Greater Yellowstone Ecosystem (GYE) in the Northern Rocky Mountains, USA, to assess the extent of climate-induced change over the recent historical period. The GYE has witnessed increased temperatures and declines in snowpack and stream flows over the last half century and these trends are projected to continue^16^,^17^. How ecosystems have responded to these recent changes is, however, largely unknown.

We used three approaches to determine how climate variation and atmospheric changes may have affected long-term dynamics of grassland production. First, we examined the degree to which production and climate are coupled by using convergent cross-mapping (CCM)^18^, a method which tests the extent to which states of time series *X* (for example, rainfall) can be predicted from historical states of the time series *Y* (for example, above-ground net primary production (ANPP)). Second, we isolated the seasonal timing and plant functional group response to potential external forcing via a 44-year snow-addition experiment^15^. We compared production in our unmanipulated grassland to production in embedded plots in which we experimentally doubled and quadrupled ambient snowpack with snow fences. Snow addition thermally buffers soils, postpones the growing season by 2 to 4 weeks and has caused shifts in plant community composition. Although control plots have remained compositionally similar over the study period, snow addition has resulted in the near complete replacement of *Festuca idahoensis* by other perennial grasses (*Bromus* and *Stipa*) and shifted the forb community from perennial species (control) to significant (× 2) and complete (× 4) dominance by annual species. We thus used ANPP in these plots to (1) control for any external forcing during winter and (2) examine potential dependency of forcing responses on functional group composition (perennial versus annual forbs). Third, we tested for expression of compensatory influences of CO_2_ and N enrichment via analysis of the C and N contents and stable isotope ratios (^13^C/^12^C and ^15^N/^14^N) of grasses and forbs.

Here, we demonstrate a sustained decline in ANPP in a native subalpine *F. idahoensis* ecosystem^19^ over the last four decades and determine increasing aridity to be the primary cause. Our analysis further indicates that changes in N deposition and atmospheric CO_2_ contributed little to the long-term pattern in ANPP.

## Results

### Long-term patterns of grassland production

**Figure 1:**
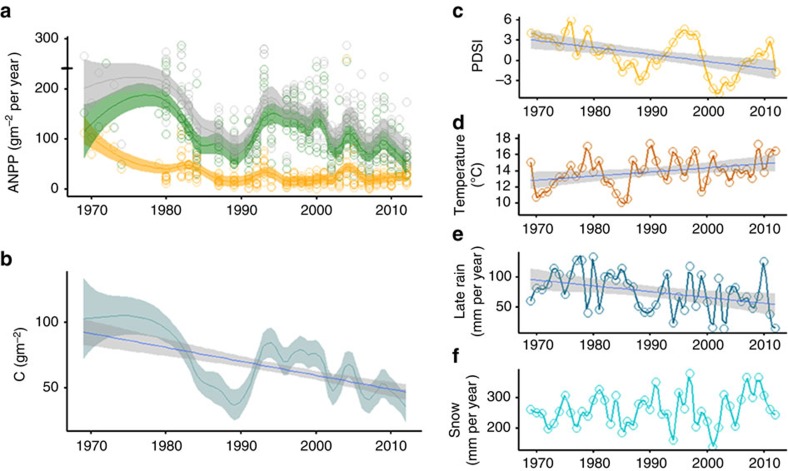
Long-term pattern of grassland production and climate variation. (**a**) Above-ground net primary production in control plots showing all plot data (points) and locally weighted trend lines with 95% confidence bands for total (grey), grass (green) and forb (orange) production. The horizontal black bar on the *y* axis is ANPP for 1965–1967 at a nearby subalpine grassland (ref. 20). (**b**) Change in total above-ground carbon pools with 95% confidence bands. (**c**) Time series of the regional Palmer Drought Severity Index (PDSI, lower values indicate increasing dryness), (**d**) September temperature, (**e**) late-summer rainfall and (**f**) annual snowfall. All significant (*P*<0.001) trends in **b**–**f** are shown with a regression line (blue) and 95% confidence intervals.

ANPP in control plots has declined by >50%, an average rate of ∼2.5 g m^−2^ yr^−2^ over the last 44 years (*P*<0001, linear regression, *n*=264; Fig. 1a, Supplementary Fig. 1) resulting in a cumulative reduction in C fixation of 1.2 kg C m^−2^ (Fig. 1b). Although the decline was continuous over the study period, yield oscillated sub-decadally, punctuated by a major dip in 1985–1992, but never exceeded early (1969–1974) levels observed at our site or those at another grassland^20^ nearby (1965–1967; Fig. 1a). Since 1995, total ANPP declined continuously at a rate equivalent to the overall (1969–2012) decline. The steep dip in ANPP in ∼1985 occurred in both C_3_ grasses and perennial forbs but only grass production recovered to the overall trend line.

### Long-term patterns in regional climate

We tested for potentially controlling changes in regional climate by analysing records from nearby weather stations and the derivative regional Palmer Drought Severity Index (PDSI, in which lower values indicate drought). Regression analysis showed that over the 1969–2012 study period, the PDSI declined significantly (*P*<0.001, *n*=44), consistent with the regional half-century drying trend (Fig. 1c). Superimposed on the trend are sub-decadal hills and troughs in PDSI values that mark multi-year droughts. Over the same period, mean annual temperature increased significantly (+0.02 °C per year) driven by increased temperatures in January (+0.08 °C per year), March (+0.05 °C per year) and September (+0.05 °C per year; Fig. 1d). Most remarkably, we report a previously undocumented >50% decline in August and September rainfall that parallels the long-term ANPP decline and increasing regional PDSI dryness (Fig. 1e, Supplementary Fig. 2) and declining late-summer stream flows^17^. No other climate variable, including snowfall (Fig. 1f) and snowpack, showed significant directional change (Supplementary Fig. 3) despite documented decreases in snowpack in the wider Northern Rockies region^16^.

### Climate control of production and plant functional groups

**Figure 2:**
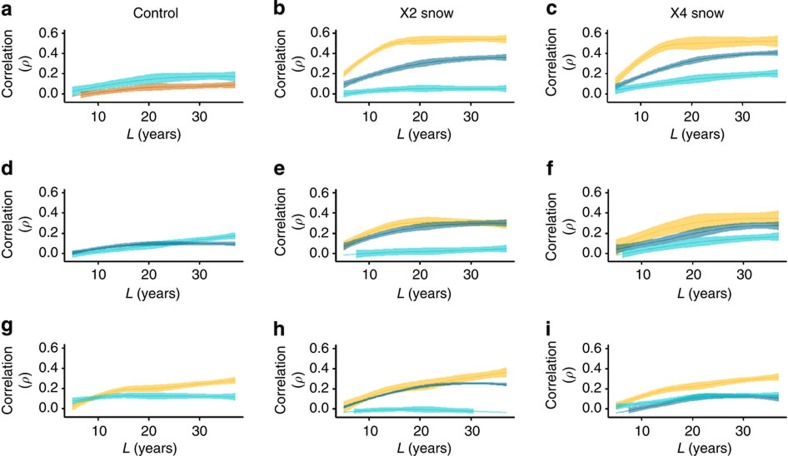
Convergent cross-mapping (CCM) results for climate forcing on ANPP across treatments. (**a**–**c**) Cross-mapping of total above-ground net primary production (ANPP) versus climate variables. (**d**–**f**) Cross-mapping of grass ANPP versus climate variables. (**g**–**i**) Cross-mapping of forb ANPP versus climate variables. Significant causal coupling of climate and ANPP is indicated by increasing correlation (*ρ*) between predicted and observed values with increasing time series length (*L*, years). Significant cross-mapping was found for snowfall (light blue), late-summer rain (dark blue), September temperature (dark orange) and the regional PDSI (light orange). Confidence bands (95%) represent uncertainty in CCM resulting from imputation of missing values.

Total ANPP was significantly (*P*<0.01, *t*-test, *n*=108 versus 156) lower (25–40%) during PDSI drought phases (PDSI values <0; 1985–1991, 2000–2007) compared with non-drought periods. ANPP was significantly but weakly correlated (Pearson's, *n*=264) with snowfall (*r*=−0.15, *P*=0.016), late-summer rain (*r*=0.26, *P*<0.0001) and temperatures (*r*=−0.19, *P*=0.0017) and the regional PDSI (*r*=0.26, *P*<0.0001) in the same year. CCM analysis, which accounts for lagged coupling of *Y* and *X* to predict current states of *X* from past states of *Y*, showed that total ANPP dynamics were driven largely by snowfall and late-summer temperatures, as indicated by the rate of increase in the estimation skill (*ρ*) of cross-mapping as the length of the time series increased (Fig. 2a). This analysis also revealed that forb and grass responses differed: grass dynamics were forced by snowfall and late-summer rain while forbs were forced by snowfall and PDSI aridity (Fig. 2d,e).

### Long-term effects of experimental snow addition

**Figure 3:**
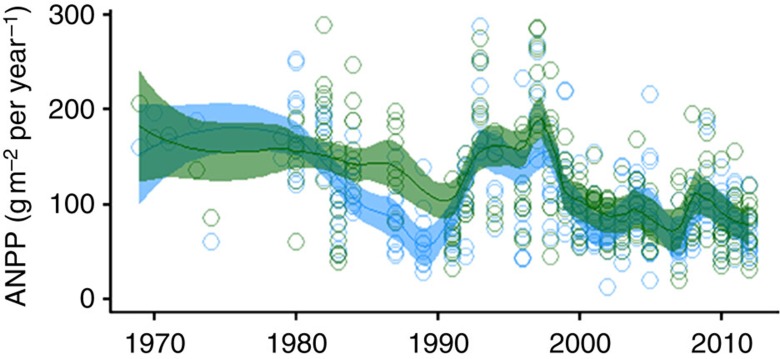
Long-term pattern of plant production in snow-addition treatments. Above-ground net primary production (ANPP) in treatments in which snowpack has been doubled (× 2, blue points and 95% confidence bands) and quadrupled (× 4, green points and 95% confidence bands) declined (*P*<0.001, linear regression, *n*=258) over time at equivalent rates as control plots.

We used a concurrent 44-year snow manipulation experiment^15^ to further identify the environmental factors forcing ANPP and the season of their action. We show that, relative to the control (−2.5 g m^−2^ yr^−2^), ANPP has declined (*P*<0.001, linear regression, *n*=258) at a similar rate in the snow augmented plots (∼2.1 and 2.7 g m^−2^ yr^−2^ in × 2 and × 4 plots, respectively; Fig. 3). The decline was CCM-driven by the PDSI and late-summer rain and only weakly coupled with snowfall (Fig. 2b,c). Although snow addition did slightly but significantly (*P*<0.01, *t*-test, *n*=258 versus 264) reduce long-term mean ANPP (110 g m^−2^ per year in × 2 and 123 g m^−2^ per year in × 4) relative to control plots (141 g m^−2^ per year), ANPP in all treatments converged to equivalently low levels by 2012 (Figs 1a and 3). Production was more strongly forced by the PDSI and late-season rain in the snow-supplemented plots, which further implies that the ANPP decline is controlled by late-summer conditions. These data also confirm that the decline in ANPP was not dependent on particular plant species or functional groups: while snow treatment replaced both grass and forb species common to control plots, production of these taxa declined at equivalent rates. Among forb species, the snow-induced replacement was from perennial to annual. While the snowpack treatments thermally buffered soils during winter, postponed the initiation of growth in the spring, had no impact on the plant–available water in surface soils (Supplementary Information; Supplementary Fig. 4) but likely postponed drying of deep (>30 cm) soil^15^, we deduce that these factors had no apparent effect. We therefore reject the hypothesis that changes in winter or immediate post-melt conditions have influenced the ANPP decline in this grassland.

### Effects of CO_2_ enrichment and nitrogen deposition

**Figure 4:**
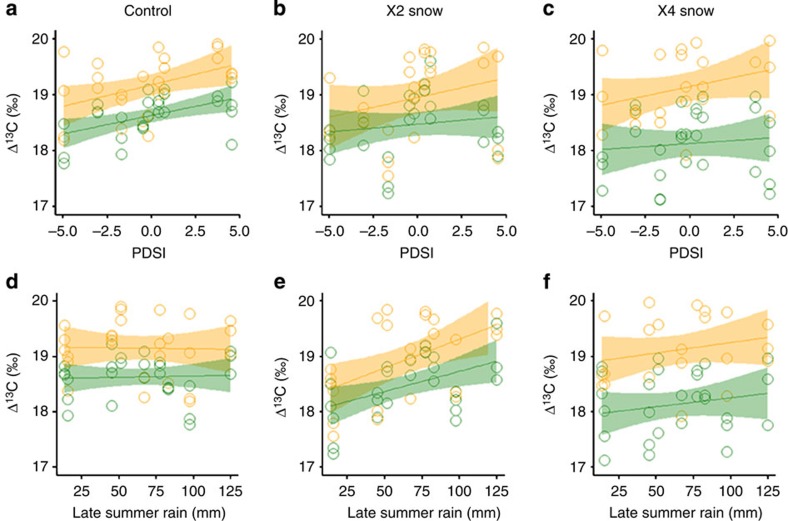
Patterns of ^13^C discrimination (Δ) across treatments and plant functional groups. (**a**–**c**) Response of Δ in grass (green points and 95% confidence region of linear regression) and forbs (orange points and 95% confidence region of linear regression) to variation in the palmer Drought Severity Index (PDSI) and (**d**–**f**) late-summer rain. Across all treatments Δ by forbs decreased significantly (*P*<0.01, linear regression, *n*=27) with decreasing PDSI values (that is, increasing PDSI drought), indicating increasing water use efficiency. The Δ by grass also decreased significantly with increasing PDSI drought in control plots (**a**). The Δ by forbs and grasses decreased significantly with decreasing late-summer rain only in × 2 treatments (**e**).

We examined the possibility that aridity effects have been understated owing to concurrent increases in atmospheric CO_2_ and N pollution, either or both of which could have resulted in compensating increases in plant production via enhanced plant water use efficiency (WUE), CO_2_ fertilization and/or increased N availability^5^,^6^,^21^. First, we find that forbs displayed consistently higher ^13^C discrimination (Δ)^22^ compared with grasses, consistent with lower WUE, irrespective of experimental treatment, or functional group dominance or species composition (Fig. 4). Treatments and associated plant groups did differ, however, in the degree to which Δ was associated with indices of dryness in a given year. In particular, Δ of forbs decreased significantly with increasing PDSI dryness across all treatments while grasses were most responsive to PDSI in control plots and summer rainfall in × 2 plots. No plant functional group or treatment showed a response of Δ to snowfall variation, further supporting late-summer control of WUE responses in this grassland. Further, we found no evidence of an overall trend in Δ (that is, from increasing CO_2_) in any treatment.

Second, it is unlikely that changes in N deposition affected the long-term trend in ANPP. That atmospheric N deposition, which has been implicated in productivity changes in other grasslands^21^,^23^, is low (<1.5 kg N ha^−1^ per year) in this region and has not increased substantially over the last three decades (Supplementary Fig. 5) argues against a contribution. Supporting this conclusion is the absence of changes in N concentrations of grasses and forbs from 1987 to 2012 (Supplementary Fig. 5). Further, biomass ^15^N, which can be a sensitive indicator of changes in the plant–soil N cycle^24^, did not show a secular change (Supplementary Fig. 5).

## Discussion

We have documented a large and persistent decline in ANPP of a terrestrial ecosystem over the last half century and attribute its cause to increasing climate dryness. Our results both complement and reinforce demonstrations of the importance of precipitation variability and seasonal timing in grasslands^7^,^9^,^25^,^26^,^27^ and interannual fluctuations in ecosystem carbon balance over large scales^2^ and extend these findings to multi-decade time scales. A critical challenge in global change research is understanding ecosystem sensitivity to long-term changes in climate, over which organisms can acclimate and communities can change via succession. Our unique long-term record allows us to distinguish ANPP responses between two prominent modes of climate variability: sub-decadal drought and multi-decadal increases in background dryness due to declining late-season rainfall and increasing temperatures. Droughts resulted in large and synchronous declines in ANPP but these were overprinted on a multi-decade decline in ANPP likely driven by increasing overall regional dryness. Less resolved were higher frequency sub-decadal oscillations in ANPP most apparent in the later record in control plots. Similar oscillations in other grasslands have been shown to result from drought-induced internal plant–soil feedbacks^25^, a possibility we have not explored here.

High sensitivity of ANPP to late-season rain was unexpected since most of the production occurs in late May and June^14^. Though snowfall is the major water input to this system and contributes to the dynamics of ANPP (as indicated by CCM in control plots), we hypothesize that, for perennial grasses and forbs, late-summer rain triggers photosynthetic gains and transfer of C to roots, from which it is applied to growth in the following spring^28^. Under drier late-summer conditions, a diminishing transfer to roots should, over time, have cascading negative consequences for future productive capacity. At the same time, we found similar ANPP declines in annual forbs, which lack this C storage capacity. Interestingly, the majority of the decline in all forbs occurred early in the record during a period of severe drought. The absence of a recovery in forb production during the succeeding moister periods, particularly in control plots, suggests that other processes such as local extinction of slowly establishing long-lived perennials or climate sensitivity of seedling establishment are operating. However, our finding of consistently higher Δ^13^C discrimination in forbs versus grasses is consistent with less conservative water use^29^ and higher drought sensitivity^30^,^31^,^32^ and likely associated with poorer regulation of water loss (stomatal control) and/or uptake (shallower rooting), supporting the idea that forbs are affected more severely by increasing dryness.

It is likely that declines in both grass and forbs (and thus overall production) would have been greater without increases in CO_2_ (∼75 p.p.m. from 1969 to 2012) and WUE. Though we discount the possibility of more than slight buffering in the ANPP decline by CO_2_ fertilization, our results indicate that any such growth stimulation was principally constrained by precipitation timing and soil water availability. Similarly, it is possible that even though N deposition has not changed significantly, interaction between climate and N cycling is contributing to the long-term ANPP decline because declining ANPP with a constant %N in biomass means that internal N turnover in the plant–soil system has also declined. Our work suggests, however, that any such feedback would be a response to changing water availability rather than an independent driver.

Our finding of declining ANPP is consistent with regionally coherent declines in water fluxes over the last half century^17^, the 2000–2009 drought-induced decline in global terrestrial NPP^3^, and regional climate–ecosystem projections over the next half century^16^. Our long-term results contrast with evidence of increased NPP under experimentally enhanced CO_2_ in other grasslands^6^,^21^ and model projections of CO_2_-driven increases in NPP in northern U.S. rangelands^33^ and suggest that changing precipitation seasonality and increasing temperatures may outpace compensatory reductions in transpirational demand. However, the extent to which our findings apply to other ecosystems across the wider region is uncertain. The degree to which our finding of climate-driven declines in ANPP are owing to recent anthropogenic perturbations to the climate system or are consistent with regional long-range natural climate variation is also unclear. Nevertheless, large and persistent climate-driven declines in C uptake over decades identifies the need to understand mechanisms that govern geographic variation in ecosystem response to anthropogenic forcing over landscape, regional and global scales.

## Methods

### Study site

Our study site lies in an extensive fescue (*Festuca idahoensis*) meadow system located on a broad flat windswept ridge (∼2,332 MASL; 45°46'59′ N, 110°46'40′ W) in the Bangtail Mountains 24 km NE of Bozeman, MT. Our study site (Bangtail Study Area, BTL) was the high elevation grassland site (‘Bridger site') for the International Biological Program studies (1964–1974) of the grassland biome^34^. Annual precipitation varies from 700 to1,000 mm, with >80% occurring as snow and the rest as rain in the June through September period. Average July maximum and minimum temperatures are 22 and 8 °C and average January maximum and minimum temperatures are −5 and −10 °C. Although the study plots have not been grazed by livestock since the 1930s, they have been accessible to the full range of large wild herbivores in the area (deer and elk). In 2007, a 1,335 ha portion of the Bangtail Ridge was established as a USDA Forest Service Special Interest Area.

### Biomass harvesting

We have harvested total end-of-season (late September to early October) above-ground standing crop in a flat 1,000 m^2^ portion of this grassland since 1969. It was harvested by clipping all vegetation in two 0.5 m^2^ quadrats randomly placed within each of five larger (200 m^2^) subplots (for a total annual harvest of *n*=10 per treatment). Specific plot locations were not resampled year-to-year. Vegetation was sorted in the field into grass and non-graminoid forb plant functional groups. We did not separate litter from current year's growth but we took care to avoid litter in the field and previous research showed that carry-over was minimal, particularly in snow treatments. Biomass samples were dried to constant weight and weighed. Early studies^14^ of vegetative production at BTL showed late growing season biomass to be a good approximation of annual ANPP although it likely represents a slight underestimate. Biomass was harvested in most years from 1969 to 2012 (exceptions were 1975–1978, 1981, 1985, 1986, 1990 and 1995) and continuously since 1996. We used mean ANPP data from the first 6 years of the study reported by Weaver and Collins^15^. We also compared ANPP during the early part of the record (1969–1974) to ANPP measured over 3 years (1965–1967) in a similar *F. idahoensis* meadow^20^ (2,408 MASL) in the GYE ∼90 km southwest of our site. In 1984, we established an independent second set of control plots 60 m northeast of our primary control area and have sampled it since, as described above. For overlapping years, both control areas show qualitatively similar dynamics (Supplementary Fig. 1).

### Experimental snow addition

A snow manipulation experiment was established at the site in 1968. The three treatments consist of control plots (described above) and snow-supplemented plots generated with snow fences (*n*=5) positioned perpendicular to prevailing winds; these double (1.2 m, ‘ × 2') and quadruple (2.4 m, ‘ × 4') ambient (<0.6 m) snow levels. Two rectangular areas with continuous snowpack roughly equivalent to the snowfence height over ∼25 m × 30 m area for the × 2 treatment and 50 m × 30 m area for the × 4 treatment resulted.

### Chemical and isotopic analysis

Biomass samples were harvested and archived for all treatments for sample years 1987, 1992, 1996, 1998, 2002, 2003, 2007, 2010, and 2012. For these years we randomly selected grass and forb samples (*n*=3–5) from each treatment and analysed them for chemical and natural abundance isotope composition (*n*=173). Forbs were not separated into annual and perennial species. The samples were homogenized, ground and analysed for %C (%), %N (%), δ^13^C (‰) and δ^15^N (‰) by isotope mass spectrometry at the Woods Hole Biological Station, MA. Biomass %C did not vary significantly (*P*>0.3) across time, between plant functional types or among treatments. We therefore applied the mean %C (44±2%) to estimate annual C stocks over time. We analysed ^13^C distributions for patterns of heavy isotope discrimination (Δ=[δ^13^C_air_−δ^13^C_plant_]/[1+δ^13^C_plant_]) as a proxy for possible changes in WUE^22^. To account for the changing ^13^C signature of atmospheric CO_2_, we used the average ^13^CO_2_ signature for June–September months spanning the 1987–2012 period recorded at La Jolla, CA and Point Barrow, AK, USA (Scripps Institution of Oceanography).

### Atmospheric nitrogen deposition

We analysed long-term patterns in regional atmospheric N inputs by first examining the long-term record of annual inorganic N deposition recorded by the National Atmospheric Deposition Program at Yellowstone Park-Tower Falls (WY08) monitoring site located 106 km south of BTL. We also examined March snowpack chemistry from three nearby sites reported by the United States Geologic Survey snow chemistry monitoring network from 1993 to 2012. We analysed data from the three nearest stations: Red Mountain (100 km due west), Big Sky (50 km southwest) and Daisy Pass (145 km south east).

### Long-term climate records

We related climate and grassland ANPP using five climate records: (1) hourly precipitation and temperature recorded (1969–2012) at the MSU weather station operated by the Optical Remote Sensor Laboratory in Bozeman (MSU; 24 km southwest of BTL); (2) hourly precipitation and temperature recorded (1969–2012) at Bozeman Yellowstone International Airport (BZN; 29 km west of BTL); (3) daily November–April snow-water equivalent recorded (1974–2012) at Bridger Bowl Ski Area (11 km northwest of BTL); (4) monthly February–May snow-water equivalent and temperature recorded (1993–2012) at Bracket Creek Snow Telemetry station (National Resources Conservation Service, United States Department of Agriculture; 13 km northwest of BTL); and (5) the regional (Bozeman area) Palmer Drought Severity Index (PDSI) for August from 1969 to 2012 (National Atmospheric and Oceanic Administration). The PDSI is based on a supply–demand algorithm of soil moisture that expresses dryness on the basis of recent precipitation and temperature and is effective at determining droughts lasting months and longer. We summarized all weather station data by month and tested for trends in annual, monthly and seasonal (winter (November–April), spring (May–June), growing season (June–September), late-summer (August–September)) values. All the weather stations are located west of BTL and thus record the effect of predominant wind direction and storms that influence BTL. For overlapping years, we evaluated the regional coherence of any secular trends via correlation between data collected at BZN (1,360 MASL) and MSU (1,500 MASL) stations and compared these with the data collected at higher elevation and closer proximity stations (Bridger Bowl: 2,026 MASL; Bracket Creek 1,890 MASL). This analysis indicates strong correspondence among weather stations (Supplementary Figs 2 and 3) suggesting that MSU and BZN records faithfully represent regionally consistent long-term climate patterns. Hence, we used the complete historical records (MSU and PDSI) for analysis ANPP–climate relationships.

### Data analysis

We applied several methods in our data analysis. To determine relationships among ANPP, climate and biogeochemical data, we used simple correlation and linear regression. We also used LOESS nonparametric regression to visualize low-frequency (4-year smoothing window) variation in biomass and associated interannual uncertainty. To identify potential causal coupling between climate and ANPP time series and to avoid inherent limitations to inferring causality using simple correlation, we applied the method of CCM^18^. CCM tests for causation by examining whether there is a signature of variable *X* in variable *Y's* dynamics by ‘cross-mapping' points in shadow *X* and *Y* manifolds constructed from lagged coordinates of *X* and *Y* time series. If *X* and *Y* are casually coupled, CCM estimation skill or predictability of cross-mapping improves with time series length (*L*), as indicated by increasing strength of correlation (*ρ*) between observed and predicted values (that is, convergence). We tested for convergence using annual means of ANPP and climate indices. To address missing values in our data (which can reduce the number of lagged-coordinate vectors used in the construction of the shadow attractor manifold), we imputed data by generating random (uniform distribution) values within limits set by the maximum and minimum observed annual biomass values occurring within a *n* +1 window on either side of the gap, where *n* is the number of years of missing data. Essentially, this generates locally weighted values and preserves trends and ‘seasonality' evident in the long-term data without imposing any prior information about climate–ANPP relationships or inferred processes. To evaluate the sensitivity of CCM results to imputed data, we generated multiple independent ANPP data sets and used them to construct ANPP–climate cross-map estimates. Only data for missing years were imputed (that is, 78% of values remained fixed within individual ANPP data sets). Cross-map estimates were robust to our imputation method as evidenced by *ρ* rapidly converging with increasing number of data sets within five runs. We therefore examined 10 data sets with imputed values for each variable (grass, forb, total) and treatment (control, × 2 snow, × 4 snow), each of which was simulated 42 times across five *L*s spanning the observation record, creating 2,100 estimates of *ρ* for each ANPP–climate cross-map analysis. We conducted CCM analyses for each driver (monthly and seasonal climate and the PDSI) and ANPP (total, grass, forbs) combination yielding a total of 1,530 independent cross-map analyses. We applied the criteria for CCM-causality as those analyses for which CCM correlations *C*_*Y X*_ were significantly higher (*P*<0.01, paired *t*-test) than those for *C*_*X Y*_. We report only CCM results that showed significant causal coupling. All statistical analyses were conducted in R (version 3.1.1)^35^.

## Additional information

**How to cite this article:** Brookshire, E.N.J. & Weaver, T. Long-term decline in grassland productivity driven by increasing dryness. *Nat. Commun.* 6:7148 doi: 10.1038/ncomms8148 (2015).

## Supplementary Material

Supplementary Information Supplementary Figures 1-5
