# Supplementary material for: Long-term decline in grassland productivity driven by increasing dryness
Source: Nat Commun. 2015 May 14;6:7148. doi: 10.1038/ncomms8148 (PMC4479003; doi:10.1038/ncomms8148)
Supplement: Supplementary Information — Supplementary Figures 1-5 [file ncomms8148-s1.pdf]

**Supplementary Figure 1**

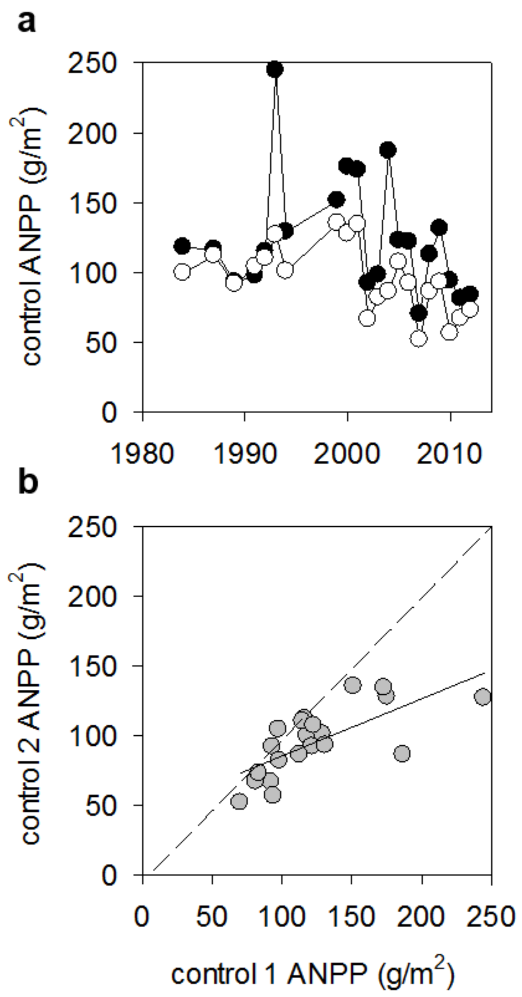


**Supplementary Fig. 1 | Similarity of long-term patterns in ANPP between control plots 1 (black symbols) and 2 (open symbols).** (a) Both control areas show significant decline ($p < 0.02$, linear regression, $n$=21) over time. (b) Simple regression between these two plots shows significant ($p < 0.001$, $n$ =21) positive temporal correspondence but overall slightly lower ANPP in control plot 2 (dashed line is 1:1) and a shallower slope driven by two high-productivity years in control 1.

**Supplementary Figure 2**

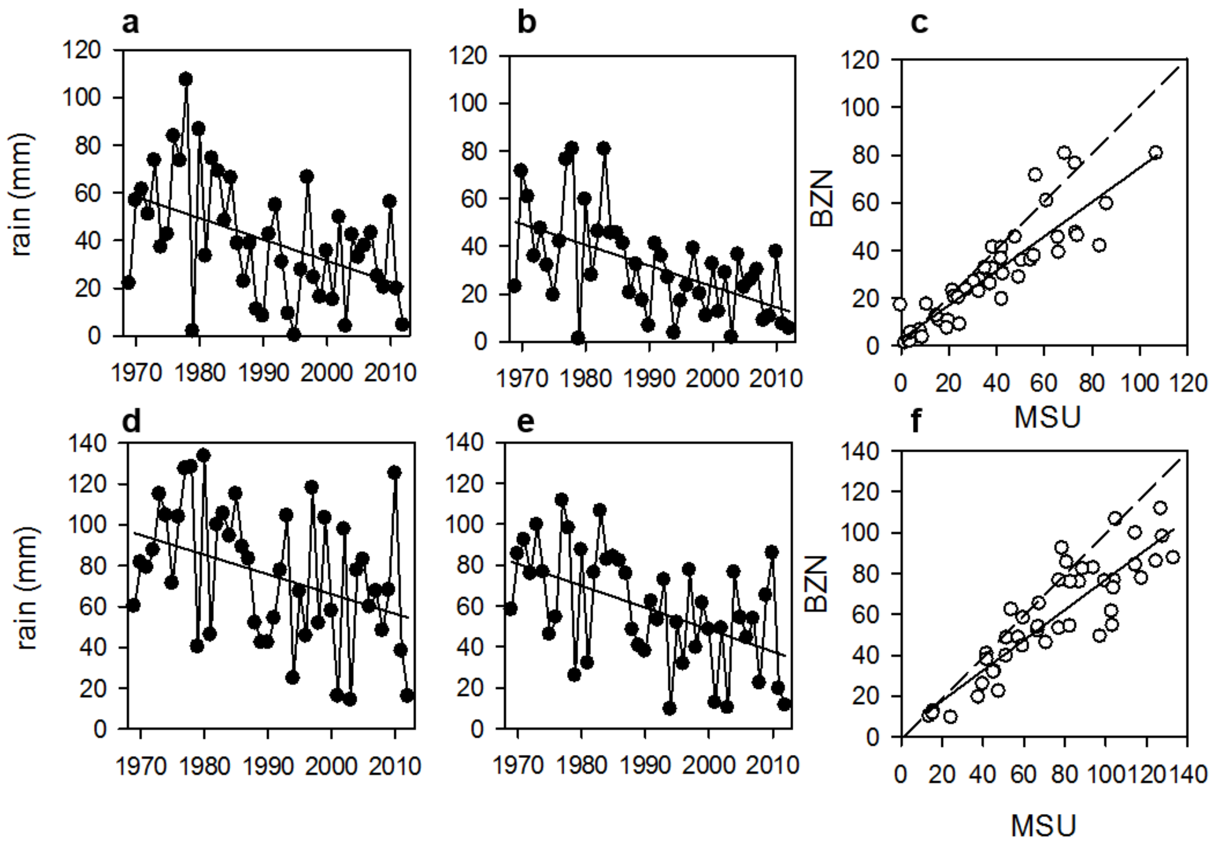


**Supplementary Fig. 2 |** Long term decline in September (a and b) and combined August and September rainfall (d and e) recorded at Montana State University (MSU; a and d) and Bozeman Yellowstone International Airport (BZN; b and e). Rainfall recorded at these stations is highly correlated ($p < 0.001$, $n = 44$) but is generally higher at MSU (c, e).

**Supplementary Figure 3**

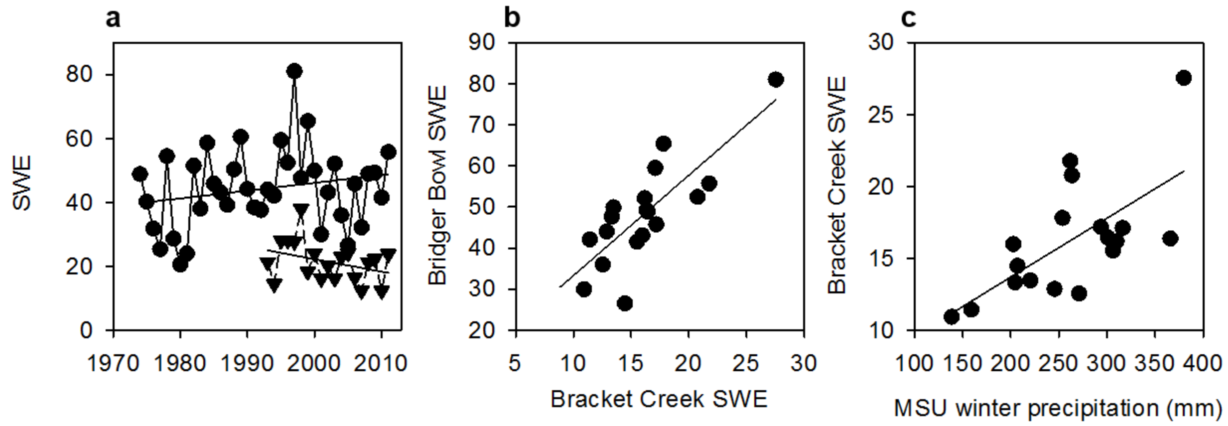


**Supplementary Fig. 3 | Time series of maximum snow water equivalent (SWE) and winter precipitation.** (a) SWE (cm) measured at Bridger Bowl (circles) and Bracket Creek SNOTEL (triangles) stations. (b) These stations show significant (*p* <0.001, linear regression, *n* =18) temporal correspondence with each other and (c) snowfall recorded at MSU, indicating strong regional coherence in winter precipitation.

**Supplementary Figure 4**

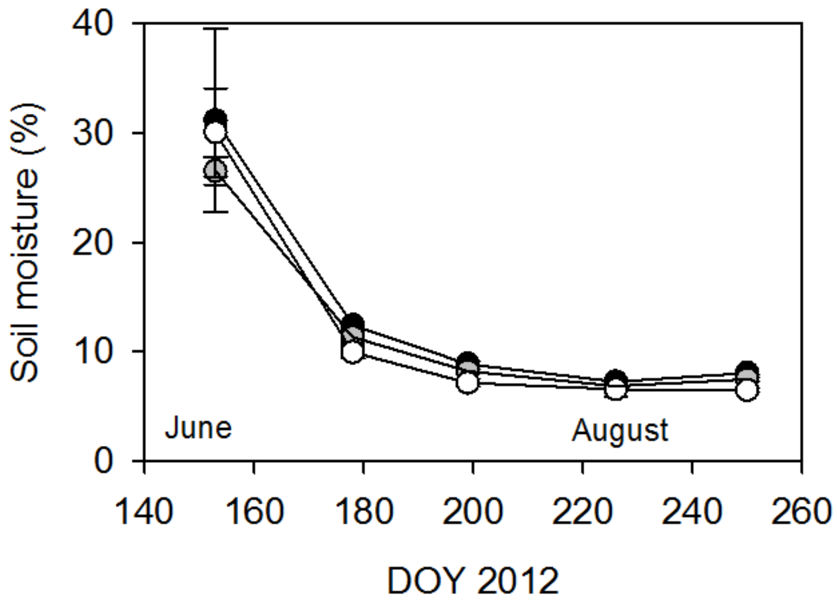


**Supplementary Fig. 4 |** Gravimetric soil moisture measured in shallow soils (0 -15 cm) as a function of day of the year (DOY) in the control (black symbols) and experimental x2-snow (grey) and x4-snow (open) plots in summer 2012. Error bars are 1SE around the mean (*n* = 5).

**Supplementary Figure 5**

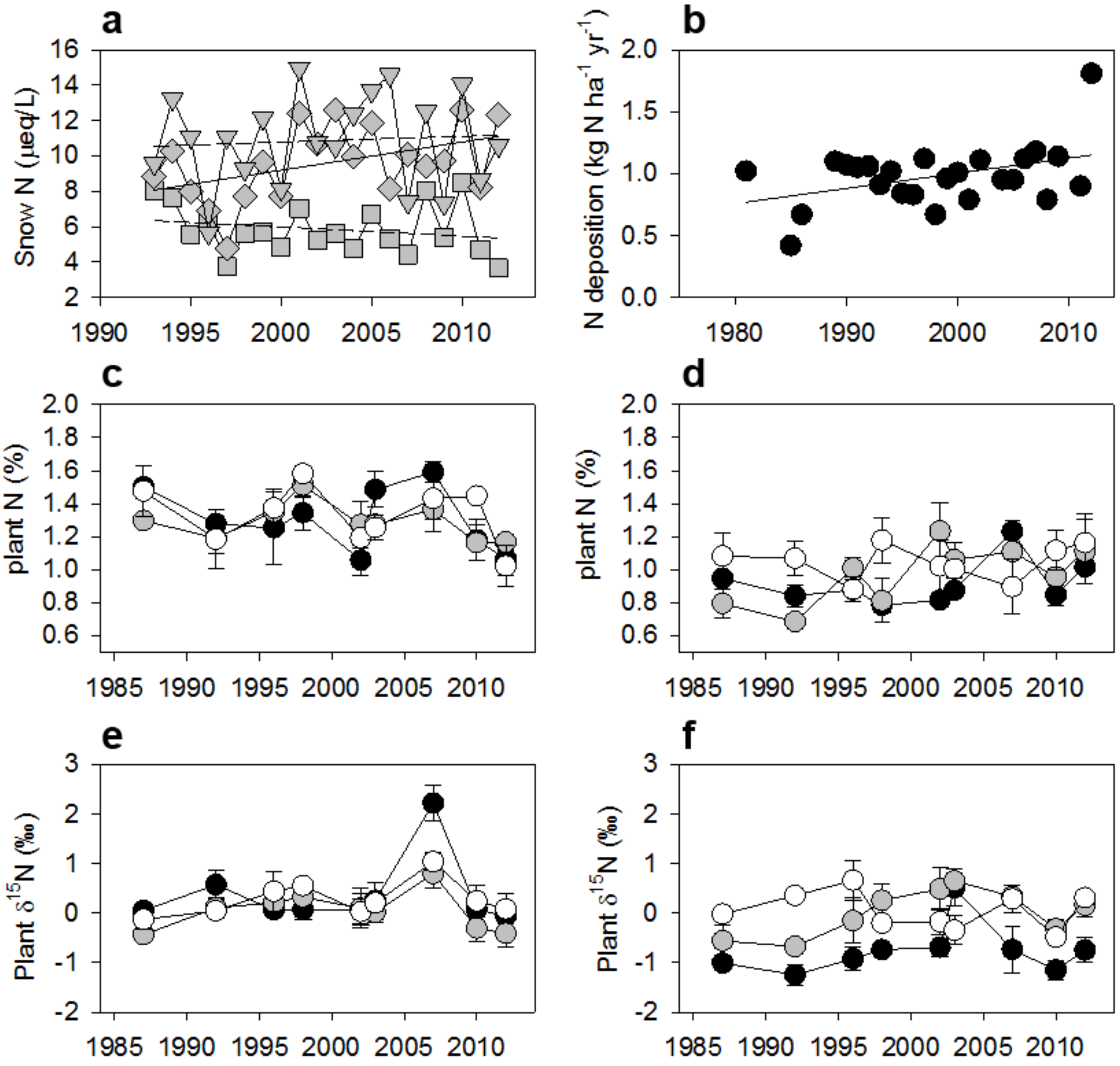


**Supplementary Fig. 5 | Time series of N inputs in precipitation and snowpack and N contents and isotopic signatures in plant biomass.** (a) N in regional snowpack and (b) atmospheric N deposition and (c and d) N contents and (e and f) $^{15}N$ composition in aboveground biomass of forbs (c and e) and grasses (d and f) across control (black symbols), x2-snow (grey symbols) and x4-snow (open symbols) plots. In (a) data from Daisy Pass (diamond symbols) showed a

significant ($P$ = 0.048) but small positive trend.  Regressions for Big Sky (squares) and Red Mountain (triangles) were not significant ($P > 0.3$).  Atmospheric N deposition is low but increased significantly ($p$ = 0.037, linear regression, $n$ = 22) at the Yellowstone NADP site. However, this relationship is rendered insignificant ($p$ = 0.2) after omitting the 2012 value. There were no significant temporal trends in N contents or $^{15}$N across functional groups and experimental treatments.  Error bars are ± 1 SE.
